# Grundzüge des neuen Genehmigungsverfahrens für klinische Arzneimittelprüfungen im Rahmen der Verordnung (EU) Nr. 536/2014 und der Zusammenarbeit zwischen den Mitgliedstaaten

**DOI:** 10.1007/s00103-022-03621-z

**Published:** 2022-12-07

**Authors:** Thomas Sudhop, Claudia Riedel, Hartmut Krafft

**Affiliations:** 1 Abteilung Informationstechnik/Klinische Prüfung, Bundesinstitut für Arzneimittel und Medizinprodukte (BfArM), Kurt-Georg-Kiesinger-Allee 3, 53175 Bonn, Deutschland; 2 Paul-Ehrlich-Institut (PEI), Langen, Deutschland

**Keywords:** Klinische Prüfung von Humanarzneimitteln, Clinical Trials Regulation (CTR), Ethik-Kommissionen, Clinical Trials Information System (CTIS), Pharmakovigilanz, Clinical trials on human medicinal products, Clinical Trials Regulation (CTR), Ethics committees, Clinical Trials Information System (CTIS), Pharmacovigilance

## Abstract

Mit der am 31.01.2022 anwendbar gewordenen Verordnung (EU) Nr. 536/2014 zu klinischen Prüfungen mit Humanarzneimitteln wurde die weitgehende Vollharmonisierung der Genehmigungs- und Überwachungsverfahren klinischer Arzneimittelprüfungen in der Europäischen Union (EU) und dem Europäischen Wirtschaftsraum (EWR) vollzogen. Neben einem vollständig papierlosen Antragsverfahren erfolgt auch die gesamte Kommunikation aller Beteiligten über das eigens für die Verordnung entwickelte *Clinical Trials Information System *(CTIS), über das auch – jeweils zeitlich gestaffelt – alle nicht geschützten Informationen und Inhalte des Genehmigungsantrags und der Ergebnisse der klinischen Prüfung der Öffentlichkeit zugänglich gemacht werden. Wie bereits unter den alten rechtlichen Rahmenbedingungen ergeht die Genehmigung einer klinischen Prüfung durch die jeweils betroffenen Mitgliedstaaten. In den Fällen, in denen eine klinische Prüfung in mehreren Mitgliedstaaten durchgeführt werden soll, erfolgt die Bewertung des allgemeinen Teils der Unterlagen nunmehr gemeinsam durch die betroffenen Mitgliedstaaten unter koordinierender Federführung eines berichterstattenden Mitgliedstaates. Der vorliegende Artikel skizziert das Genehmigungsverfahren mit seinem Fristenkonzept und adressiert weitere Aspekte der Verordnung, wie z. B. Details zum Schutz der an der klinischen Prüfung teilnehmenden Personen, die Sicherheitsberichterstattung sowie die Transparenzregelungen.

## Abstract (en)

With Regulation (EU) No. 536/2014 on clinical trials on medicinal products for human use, which became applicable on 31 January 2022, full harmonisation of the authorisation and monitoring procedures of clinical trials with medicinal products in the European Union (EU) and the European Economic Area (EEA) has been achieved. In addition to an entirely paperless application procedure, communication between all parties involved is done through the *Clinical Trials Information System *(CTIS), which was developed specifically for the Regulation and through which all non-proprietary information and content of the clinical trial application and results are also made available to the public. As was already the case under the old legal framework, the authorisation of a clinical trial is granted by each Member State concerned; however, the assessment of the common part of the dossier of a clinical trial that is conducted in more than one Member State is jointly done by the respective Member States under the coordinating lead of a reporting Member State. The present article outlines the authorisation procedure with its deadline concept and addresses further aspects of the Regulation, such as details on the protection of the trial subjects, safety reporting and transparency rules.

## Einleitung

**Figure 1:**
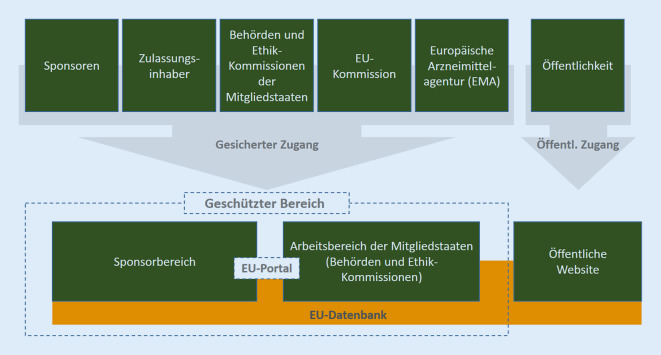

Zum 31.01.2022 wurde die „Verordnung (EU) Nr. 536/2014 des Europäischen Parlaments und des Rates vom 16. April 2014 über klinische Prüfungen mit Humanarzneimitteln und zur Aufhebung der Richtlinie 2001/20/EG“ erstmals anwendbar [1]. Vorausgegangen war eine mehr als 7‑jährige Entwicklungszeit für die in Kapitel XIV der Verordnung vorgesehene IT-Infrastruktur. Diese bildet die Grundlage für die ausschließlich elektronische Antragstellung und das Genehmigungsverfahren einschließlich der gesamten Kommunikation zwischen allen Beteiligten. Startschuss für die Anwendbarkeit der Verordnung war die Inbetriebnahme des „Clinical Trials Information System“ (CTIS), welches im Wesentlichen das EU-Portal und die EU-Datenbank gemäß Artikel 80 und 81 der Verordnung (Abb. 1; [2]) umfasst und welches von der Europäischen Arzneimittelagentur (EMA) unter Mitarbeit der Mitgliedstaaten entwickelt und am 31.01.2022 in den Wirkbetrieb genommen wurde [3]. Der vorliegende Artikel soll die grundlegenden Elemente und Prinzipien der Verordnung, insbesondere das neue gemeinsame Bewertungsverfahren, Übergangsregelungen, Rollen und Kernelemente der Verordnung, Sicherheitsbewertung und Transparenz, sowie weitere wesentliche, neu hinzugekommene Verfahren beleuchten.

## Übergangsregelungen

Obwohl die Verordnung zum 31.01.2022 anwendbar wurde und damit z. B. auch alle Definitionen in Artikel 2 der Verordnung die bisherigen Definitionen ersetzten, sieht die Verordnung in den Übergangsbestimmungen in Artikel 98 vor, dass klinische Prüfungen, für die ein Genehmigungsantrag gemäß der Richtlinie 2001/20/EG [4] bis zum 30.01.2023 eingereicht wird, noch bis zum 30.01.2025 im alten Rechtskontext fortgeführt werden. Daher sind für solche klinischen Prüfungen in Deutschland das Arzneimittelgesetz (AMG; [5]) und die GCP-Verordnung^1^ [6] in der jeweiligen bis zum 26.01.2022 geltenden Fassung anzuwenden. Eine entsprechende Präzisierung dazu findet sich in § 148 AMG. Alle bis zum 30.01.2025 noch laufenden klinischen Prüfungen müssen bis dahin in den Rechtskontext der Verordnung überführt worden sein oder ihre Genehmigung erlischt. Für klinische Prüfungen mit Arzneimitteln, die nicht in den Anwendungsbereich der Verordnung fallen, z. B. bestimmte Gewebezubereitungen, sind gemäß § 148 Abs. 3 AMG das Arzneimittelgesetz und die GCP-Verordnung in der jeweils am 26.01.2022 geltenden Fassung noch bis zum 23.12.2029 weiter anzuwenden.

## Kernelemente der Verordnung

Durch die Änderung des Rechtsrahmens der Richtlinie 2001/20/EG, die in allen Mitgliedstaaten im Jahr 2004 in nationales Recht implementiert wurde, in eine unmittelbar in allen Mitgliedstaaten gültige und anwendbare Europäische Verordnung wurde eine weitgehende Vollharmonisierung des Genehmigungs- und Überwachungsverfahrens klinischer Prüfungen von Humanarzneimitteln in der Europäischen Union (EU) und dem Europäischen Wirtschaftsraum (EWR) vollzogen. Wie bereits in der vorhergehenden Richtlinie ist vor Beginn der Durchführung einer klinischen Prüfung eine Genehmigung durch die zuständigen Stellen eines betroffenen Mitgliedstaats erforderlich, d. h., das Erfordernis einer nationalen Genehmigung bleibt auch unter der Verordnung erhalten.

Allerdings erfolgt nach der neuen Verordnung die Bewertung der Unterlagen bei multinationalen klinischen Prüfungen parallel gemeinsam in allen beteiligten Mitgliedstaaten (*Member States Concerned, MSC*), in denen die klinische Prüfung durchgeführt werden soll. Zur Koordinierung und Verfahrensführung wird unter den jeweils beteiligten Mitgliedstaaten ein berichterstattender Mitgliedstaat (*Reporting Member State, RMS*) festgelegt. Dieser koordiniert das Verfahren, harmonisiert mögliche Einwände und Nachfragen und ist für die Fertigung des allgemeinen Teils *(Teil I) des Bewertungsberichts* verantwortlich (s. unten). Die Ermittlung des RMS erfolgt in den ersten 6 Tagen nach Antragstellung. Dabei hat der Sponsor ein Vorschlagsrecht, d. h., er schlägt unter allen beteiligten Mitgliedstaaten für einen Genehmigungsantrag einen Mitgliedstaat vor, den er sich als RMS wünscht. Die endgültige Entscheidung wird anhand des Vorschlags, der vorhandenen Kapazitäten und bisherigen Auslastungen unter den beteiligten Mitgliedstaaten getroffen.

Wie bereits unter der Richtlinie 2001/20/EG erfolgt die Genehmigung einer klinischen Prüfung auf der Basis der Prüfung von bestimmten Unterlagen, wie z. B. des Prüfplans oder des Dossiers zu den Prüfpräparaten. Durch die Verordnung verpflichtend ist die Erstellung eines sogenannten Bewertungsberichts (*Assessment Report, AR*), in dem die Bewertung der verschiedenen Unterlagen durch die bewertenden Personen (Assessoren) dokumentiert wird. Für jede klinische Prüfung wird ein Bewertungsbericht durch den RMS unter Zuarbeit der MSC beim Erstantrag erstellt, der ggf. im weiteren *Life Cycle* der klinischen Prüfung überarbeitet wird.

Nicht alle Elemente einer klinischen Prüfung sind in allen Mitgliedstaaten identisch geregelt und können durch die Verordnung vollständig harmonisiert werden. So sind z. B. Regelungen zu Schadensersatz und auch die Anforderung an die Erfahrung und Qualifikation von Prüfern in einer klinischen Prüfung in Teilen unterschiedlich geregelt und unterliegen nicht den harmonisierten Rechtsbereichen der EU/EWR. Diejenigen Elemente und Dokumente einer klinischen Prüfung, die rein nationale Belange betreffen, werden im *Teil II des Bewertungsberichts* behandelt und umfassen die in Tab. 1 aufgeführten Elemente. Jeder beteiligte Mitgliedstaat erstellt den Teil II des Bewertungsberichts auf der Basis der für den beteiligten Mitgliedstaat eingereichten Unterlagen und der entsprechenden nationalen Erfordernisse.

**Table 1:** 

Aspekt	Bewertungsinhalte
Einwilligung nach Aufklärung (*Informed Consent*)	*Einhaltung der Voraussetzungen für die Aufklärung und Einwilligung in Bezug auf*
	Artikel 28 (allgemeine Grundsätze, Widerrufsrecht etc.)
	Artikel 29 (Einwilligung nach Aufklärung)
	Artikel 30 (Cluster-Prüfungen, in Deutschland nicht zutreffend)
	Artikel 31 (klinische Prüfungen mit nicht einwilligungsfähigen Prüfungsteilnehmern (Minderjährige und Erwachsene)), zusätzlich auch § 40b Arzneimittelgesetz (AMG)
	Artikel 32 (klinische Prüfungen mit Minderjährigen)
	Artikel 33 (klinische Prüfungen mit schwangeren oder stillenden Frauen)
	Artikel 34 (zusätzliche nationale Einschränkungen, in Deutschland § 40a Satz 1 Nr. 2 AMG)
	Artikel 35 (klinische Prüfungen in Notfällen)
Aufwandsentschädigung für Prüfungsteilnehmer und Prüfer	Verbot von Anreizen für Prüfungsteilnehmer gemäß Artikel 28, 31, 32 und 33
	Angaben zu Finanzierung gemäß Anhang I, Buchstabe P
Rekrutierung von Prüfungsteilnehmern	Übereinstimmung mit den Anforderungen in Kapitel V in Bezug auf die Rekrutierung
	Angaben zur Rekrutierung in Anhang I, Buchstabe K
Datenschutz für Prüfungsteilnehmer	Übereinstimmung mit den Anforderungen der Datenschutzgrundverordnung (DSGVO)
Eignung der Prüfer und Mitglieder des Prüfungsteams	Übereinstimmung mit den Vorgaben in Artikel 49 zur Eignung der an der Durchführung einer klinischen Prüfung mitwirkenden Personen
Eignung der Prüfstelle(n)	Übereinstimmung mit den Vorgaben in Artikel 50 zur Eignung der Einrichtungen, in denen eine klinische Prüfung durchgeführt werden soll
Regelungen zum Schadensersatz	Übereinstimmung mit den Vorgaben in Artikel 76 zu Schadensersatzregelungen i. V. m. § 40a Satz 1 Nr. 3 AMG
Biologische Proben	Übereinstimmung mit den Bestimmungen über die Gewinnung, Lagerung und zukünftige Nutzung der vom Prüfungsteilnehmer genommenen biologischen Proben

Alle übrigen Dokumente und Aspekte werden im *Teil I des Bewertungsberichts* bewertet und umfassen als wesentliche Elemente:

- den Prüfplan (*Trial Protocol*),
- die Prüferinformation (*Investigator’s Brochure, IB*),
- das Prüfpräparate-Dossier (*Investigational Medicinal Product Dossier, IMPD*),
- eventuell Unterlagen zu Hilfspräparaten (*Auxilliary Medicinal Products, AxMPs*) und
- ggf. weitere Dokumente, wie z. B. das pädiatrische Prüfkonzept (*Paediatric Investigation Plan, PIP*).

Die Angaben zu Teil I des Bewertungsberichts werden gemeinsam von allen beteiligten Mitgliedstaaten unter koordinierender Federführung des RMS bewertet und harmonisiert, d. h., die in Teil I des Bewertungsberichts bewerteten Aspekte sind für alle beteiligten Mitgliedstaaten identisch. Sofern der Sponsor keinen Antrag nach Artikel 11 der Verordnung stellt, bei dem zunächst nur die Inhalte des Teils I bewertet werden, werden Teil I und Teil II des Bewertungsberichts gleichzeitig gestartet. Sie können aber je nach erforderlichen Nachfragen in den MSC zu unterschiedlichen Zeitpunkten abgeschlossen werden (s. unten).

## Rollen in klinischen Prüfungen

Wie bereits unter der Richtlinie trägt auch unter der Verordnung der *Sponsor* einer klinischen Prüfung die Verantwortung für die Einleitung, das Management sowie die Aufstellung der Finanzierung einer klinischen Prüfung. Dabei kann der Sponsor eine natürliche Person, ein Unternehmen, eine Einrichtung oder auch eine Organisation sein. Zusätzlich erlaubt die Verordnung eine gemeinsame Co-Sponsorschaft mehrerer Sponsoren für eine klinische Prüfung unter bestimmten Verantwortungsabgrenzungen für essentielle Aufgaben, wie z. B. das Genehmigungsverfahren oder die Sicherheitsberichterstattung. Als *Prüfer* wird eine für die Durchführung einer klinischen Prüfung an einer Prüfstelle verantwortliche Person bezeichnet, als *Hauptprüfer* der verantwortliche Leiter eines Prüferteams an einer *Prüfstelle*.

Während die Richtlinie noch eine Unterscheidung zwischen nationaler zuständiger Behörde und den nationalen Ethikkommissionen vorsah, ist diese Trennung in der Verordnung nicht mehr definiert. Gemäß der Verordnung werden die Entscheidungen zu einer klinischen Prüfung durch den jeweiligen Mitgliedstaat getroffen; durch welche Körperschaft dies erfolgt, ist dem jeweiligen Mitgliedstaat vorbehalten und nicht einheitlich in der EU/EWR geregelt. Die Verordnung geht zwar in jedem Fall von einer Mitwirkung von Ethikkommissionen bei der Bewertung von klinischen Prüfungen aus. Allerdings wurde die sehr unterschiedliche Beteiligung von Ethikkommissionen in den einzelnen Mitgliedstaaten in Bezug auf deren Mitwirkung auch bei der Bewertung von Teil I und nicht nur von Teil II bereits schon vor Anwendbarkeit der Verordnung intensiv und kontrovers diskutiert [7]. Umgekehrt kann eine Ethikkommission – sofern die nationale Gesetzgebung des Mitgliedstaates dies vorsieht – sogar ein Vetorecht ausüben; in Deutschland ist dies nur bedingt in Bezug auf die Bewertung der Inhalte des Teils II des Bewertungsberichts vorgesehen, die ausschließlich durch die zuständige Ethikkommission vorgenommen wird.

## Das Genehmigungsverfahren

### Grundprinzipien der Fristen im Genehmigungsverfahren

Die Fristen im Genehmigungsverfahren der Verordnung sind generell kürzer als noch unter der Richtlinie und beinhalten das Prinzip des „tacit withdrawal“ bzw. „tacit approval“. Dies bedeutet, dass, wenn ein antragstellender Sponsor eine vom berichterstattenden – oder in Bezug auf Teil II des Bewertungsberichts vom jeweils betroffenen – Mitgliedstaat angeforderte Information nicht innerhalb der gesetzten Frist über CTIS zur Verfügung stellt, der Antrag implizit und automatisch als hinfällig gilt. Damit ist das Antragsverfahren in allen beteiligten Mitgliedstaaten beendet, wenn dies aufgrund von Inhalten den Teil I des Bewertungsberichts betreffend eintritt. Bei Elementen, die ausschließlich den Teil II des Bewertungsberichts betreffen, ist der Antrag jeweils nur im betroffenen Mitgliedstaat hinfällig. Umgekehrt gelten bestimmte Teilschritte des Antragverfahrens als implizit positiv bewertet, wenn der jeweils verantwortliche Mitgliedstaat nicht innerhalb der in der Verordnung vorgesehenen Frist seine Stellungnahme bzw. Entscheidung über CTIS mitteilt. Dieses Prinzip gilt sowohl für das Verfahren der Erstgenehmigung, für nachfolgende wesentliche Änderungen wie auch das Verfahren zum Hinzufügen weiterer Mitgliedstaaten. Aus Platzgründen sind im Nachfolgenden nur die Fristen für ein Erstgenehmigungsverfahren dargestellt.

Sowohl das Erstgenehmigungsverfahren wie auch die Folgegenehmigungsverfahren wesentlicher Änderungen einer bereits genehmigten klinischen Prüfung verlaufen in der Regel 3‑phasig und umfassen eine Validierungsphase, eine Phase der (gemeinsamen) inhaltlichen Bewertung und eine nationale Entscheidungsphase.

In der Phase der *Validierung*, die maximal 25 Tage betragen darf, wird der Antrag auf Vollständigkeit der vorzulegenden Unterlagen und Informationen von allen beteiligten Mitgliedstaaten gesichtet und auch geprüft, ob die beantragte klinische Prüfung in den Geltungsbereich der Verordnung fällt. Fehlen Unterlagen oder relevante Angaben, so werden diese bis maximal Tag 10 beim Antragsteller nachgefordert. Erfolgt die Nachlieferung nicht innerhalb der gesetzten Frist von maximal 10 Tagen, gilt der Antrag als hinfällig.

In der sich nach erfolgreicher Validierung anschließenden *Bewertungsphase* (Assessment Phase) erfolgt die inhaltliche Bewertung, für Inhalte den Teil I betreffend gemeinsam mit allen MSC unter Federführung und Koordinierung des RMS, für Inhalte bzgl. Teil II jeweils ausschließlich in den MSC. Das Bewertungsverfahren umfasst bei multinationalen klinischen Prüfungen 3 Phasen:

1. die Phase der Erstbewertung durch den RMS mit Erstellung eines Entwurfs des Teils I des Bewertungsberichts (maximal 26 Tage),
2. die anschließende Phase der koordinierten Überprüfung durch alle MSC auf der Grundlage des übersandten Entwurfs (maximal 12 Tage) und
3. die abschließende Phase der Konsolidierung (maximal 7 Tage), die vom RMS vorgenommen wird und in der er die endgültige Fassung des Teils I des Bewertungsberichts unter gebührender Berücksichtigung der Anmerkungen der MSC erstellt.

Diese insgesamt *45 Tage* für die Bewertung des Teils I können vom RMS um *weitere 31 Tage verlängert* werden, wenn Nachfragen an den Sponsor erforderlich sind. Diese „requests for information“ (RFI) müssen innerhalb der dem Sponsor gesetzten Frist, die maximal 12 Tage betragen kann, beantwortet werden. Unmittelbar nach Eingang der Sponsorantwort in CTIS wird diese von allen MSC innerhalb von 12 Tagen bewertet und der RMS finalisiert den Teil I des Bewertungsberichts einschließlich der Schlussfolgerung zur Genehmigung der Inhalte des Teils I innerhalb weiterer 7 Tage, so dass spätestens an *Tag 76* nach Validierung eine Entscheidung zu Teil I dem Sponsor und den übrigen MSC vom RMS über CTIS mitgeteilt wird.

Das Verfahren für die Erstbewertung zu Teil II startet – sofern das hier nicht näher beschriebene Verfahren nach Artikel 11 zur Anwendung kommt – ebenfalls mit dem Tag der Einreichung über CTIS. Für die Inhalte zu Teil II ist keine vorgeschaltete explizite Validierung vorgesehen. Fehlende Unterlagen werden im Verfahren vom jeweiligen Mitgliedstaat nachgefordert. Jeder Mitgliedstaat fertigt den Bewertungsbericht zu Teil II innerhalb von maximal 45 Tagen und übermittelt diesen einschließlich der Schlussfolgerung via CTIS an den Sponsor. Jeder betroffene Mitgliedstaat kann innerhalb dieser 45 Tage vom Sponsor zusätzliche Informationen zu Teil II anfordern, die der Sponsor innerhalb von höchstens 12 Tagen nachliefern muss. Dem jeweiligen Mitgliedstaat stehen ab dem Zeitpunkt des Eingangs der Nachlieferung in CTIS maximal weitere 19 Tage zur Verfügung, um den Teil II des Bewertungsberichts zu finalisieren, so dass sich bei Ausnutzung aller Fristen die Zeit für die Erstellung des Teils II des Bewertungsberichts auch hier von *45 auf 76 Tage* verlängert.

Am Ende der Frist für die Bewertungsphase schließt sich dann die *(nationale) Entscheidungsphase* an, in der jeder beteiligte Mitgliedstaat dem Sponsor auf dem Wege einer nationalen Mitteilung bzw. Bescheids mitteilt, ob er die klinische Prüfung genehmigt, mit Auflagen genehmigt oder die Genehmigung versagt. Grundlage für die Entscheidung sind die Aspekte in Teil I und Teil II des Bewertungsberichts. Für die Entscheidung in Bezug auf Teil I des Bewertungsberichts kommt dem RMS eine entscheidende Rolle zu: Grundsätzlich müssen alle MSC der Entscheidung des RMS zu Teil I des Bewertungsberichts folgen. Im Falle einer Versagung ist diese Entscheidung für alle MSC zwingend bindend. Für den Fall einer Genehmigung zu Teil I, ggf. auch unter Auflagen, kann ein MSC in wenigen Einzelfällen von dieser positiven Entscheidung des RMS abweichen („opt out“). Ein Abweichen ist nur dann möglich, wenn:

- der MSC der Auffassung ist, dass die Teilnahme an der klinischen Prüfung dazu führen würde, dass Prüfungsteilnehmer eine schlechtere Behandlung erhalten würden, als dies gemäß normaler klinischer Praxis in dem betroffenen Mitgliedstaat der Fall wäre,
- ein Verstoß gegen bestimmte, in Artikel 90 der Verordnung genannte nationale Rechtsvorschriften^2^ vorliegt oder
- der MSC Bedenken hinsichtlich der Sicherheit der Prüfungsteilnehmer sowie der Zuverlässigkeit und Belastbarkeit der übermittelten Daten bzw. Informationen hat.

Ist ein MSC der Auffassung, dass ein solcher Opt-out-Grund vorliegt, versagt er die Genehmigung der klinischen Prüfung; dies gilt auch, wenn er in hinreichend begründeten Fällen zu dem Schluss gelangt, dass die in Teil II des Bewertungsberichts behandelten Aspekte nicht eingehalten werden. Ergänzend entfällt ebenfalls eine nationale Genehmigung eines beteiligten Mitgliedstaates, wenn der betreffende Mitgliedstaat fehlende oder weitere Informationen zu den Inhalten des Teils II anfordert und der Sponsor diese Information innerhalb der gesetzten Frist nicht vorgelegt hat. In solchen Fällen gilt der Antrag im entsprechenden Mitgliedstaat als hinfällig.

## Hinzufügen weiterer Mitgliedstaaten

Ein Sponsor kann das Hinzufügen weiterer Mitgliedstaaten zu einer bereits genehmigten klinischen Prüfung beantragen. Hierzu sieht die Verordnung ein geringfügig verlängertes Genehmigungsverfahren vor, dass in Bezug auf die Aspekte zu Teil I durch den für die Erstgenehmigung verantwortlichen RMS koordiniert wird. Neue MSC erhalten den Bewertungsbericht und können Anmerkungen zu Teil I über CTIS an alle beteiligten Mitgliedstaaten übermitteln. Weitere Informationen vom Sponsor darf auch hier nur der RMS anfordern. Ansonsten folgen die Verfahrensgrundsätze dem Verfahren der Erstgenehmigung.

## Wesentliche Änderungen

Wesentliche Änderungen einer bereits genehmigten klinischen Prüfung einschließlich des Hinzufügens weiterer Prüfzentren oder Änderung eines Hauptprüfers bedürfen ebenfalls der vorherigen Genehmigung. Dabei gleichen die Verfahren dem Verfahren der Erstgenehmigung über CTIS mit geringfügig verkürzten Fristen. Auch hier erfolgt eine gemeinsame koordinierte Bewertung der Änderungen den Teil I des Bewertungsberichts betreffend unter Federführung des bereits für die Erstbewertung verantwortlichen RMS. Aspekte, die den Teil II des Bewertungsberichts betreffen, werden ausschließlich durch den betroffenen Mitgliedstaat bewertet. Auch bei wesentlichen Änderungen, die den Teil I des Bewertungsberichts betreffen, müssen die beteiligten Mitgliedstaaten der Schlussfolgerung des RMS folgen. Genau wie bei der Erstgenehmigung darf ein MSC von einer positiven Schlussfolgerung zu Teil I nur abweichen, wenn die oben genannten Opt-out-Gründe vorliegen. Die Bewertung wesentlicher Änderungen in Bezug auf Teil II verläuft in Analogie zur Erstbewertung mit ebenfalls geringfügig verringerten Fristen. Grundsätzlich können wesentliche Änderungen nur eingereicht werden, wenn alle anderen regulatorischen Aktivitäten, die einer Genehmigung bedürfen, im jeweils betroffenen Mitgliedstaat abgeschlossen sind. Nur isolierte Änderungen zu Teil II des Bewertungsberichts können parallel in verschiedenen Mitgliedstaaten eingereicht werden.

## Kommunikation im Genehmigungsverfahren

Die gesamte Kommunikation im Antragsverfahren soll über CTIS erfolgen. CTIS verlangt eine vorherige Anmeldung im *Organisation Management System* (OMS) der EMA, um sich gegenüber CTIS als berechtigte Person oder Institution zu identifizieren und zu autorisieren. Über ein komplexes Rollen- und Rechtesystem mit annähernd 50 Rollen und entsprechend fein granulierten Rechten können verschiedene Personen oder Gruppen des Sponsors Teile eines Antragsprozesses vorbereiten und über das System einreichen. Durch die vorherige Autorisierung und Vergabe der Rollen innerhalb einer Sponsororganisation sind in der Regel bei der Antragstellung keine Unterschriften erforderlich.

Die gesamte Kommunikation mit dem Sponsor zu Teil I erfolgt ausschließlich über den RMS, d. h., nur dieser darf RFI an den Sponsor richten. Nur bei Nachforderungen zu Teil II des Bewertungsberichts darf der betroffene MSC diese direkt über CTIS an den Sponsor richten.

Nachfragen zu Teil I, die die MSC im Laufe der Validierung und der koordinierten Bewertung haben, werden zunächst als sogenannte *Considerations* in CTIS prozessiert. Gleichgelagerte Considerations werden vom RMS konsolidiert und per CTIS dann als RFI dem Sponsor mit Fristsetzung übermittelt.

Nach Abschluss der Bewertungsphase wird dem Sponsor der Bewertungsbericht mit der Schlussfolgerung des RMS zu Teil I über CTIS übermittelt. Ist diese positiv, d. h., liegt eine (ggf. auch beauflagte) Genehmigung zu Teil I vor, fügt jeder Mitgliedstaat seine Schlussfolgerung zu Teil II hinzu und erteilt dann – sofern die Schlussfolgerung zu beiden Teilen positiv ist – eine Genehmigung in Form eines nationalen Genehmigungsbescheids. Auflagen gestattet die Verordnung nur, wenn diese ihrer Art nach nicht schon zum Zeitpunkt der Genehmigung hätten erfüllt sein können.

## Schutz der Prüfungsteilnehmer und Einwilligung nach Aufklärung

Dem Schutz der Prüfungsteilnehmer und den Verfahren zur Einwilligung nach Aufklärung (Informed Consent) ist in der Verordnung ein eigenes Kapitel mit 8 Artikeln gewidmet. Grundsätzlich orientiert sich die Verordnung an den Grundsätzen der Guten Klinischen Praxis (GCP; [8]) und den Grundzügen der Deklaration von Helsinki [9]. Eine Teilnahme an einer klinischen Prüfung erfordert in der Regel eine schriftliche Zustimmung nach vorheriger Aufklärung in einem Gespräch. Neu ist das explizite Erfordernis, dass im Aufklärungsgespräch sicherzustellen ist, dass die Prüfungsteilnehmer die Informationen verstanden haben.

Grundsätzlich muss eine klinische Prüfung so geplant sein, dass sie „mit möglichst wenig Schmerzen, Beschwerden, Angst und allen anderen vorhersehbaren Risiken für die Prüfungsteilnehmer verbunden ist und sowohl die Risikoschwelle als auch das Ausmaß der Belastung im Prüfplan eigens definiert und ständig überprüft werden“.

Für klinische Prüfungen an nicht einwilligungsfähigen Personen sieht die Verordnung besondere Voraussetzungen vor. Grundsätzlich sind auch solche Prüfungsteilnehmer ihren Fähigkeiten entsprechend angemessen aufzuklären; die Einwilligung zur Teilnahme hat jedoch durch den zuvor aufgeklärten gesetzlichen Vertreter zu erfolgen. Lassen nicht einwilligungsfähige Prüfungsteilnehmer, die in der Lage sind, sich eine Meinung zu bilden und die Aufklärungsinformationen beurteilen können, erkennen, dass sie nicht an der klinischen Prüfung teilnehmen oder ihre Teilnahme in einer laufenden klinischen Prüfung beenden wollen, so muss dies vom Prüfer beachtet werden.

Neben einem zwingenden methodischen Erfordernis zum Einschluss nicht einwilligungsfähiger Personen gilt, dass eine solche klinische Prüfung entweder einen direkten Nutzen für die Prüfungsteilnehmer haben muss, der die Risiken und Belastungen überwiegt, oder – im Falle einer ausschließlich gruppennützigen klinischen Prüfung^3^ – diese nur im „direkten Zusammenhang mit dem lebensbedrohlichen oder zu Invalidität führenden klinischen Zustand steht, unter dem der Prüfungsteilnehmer leidet, und sofern die Prüfung den betroffenen nicht einwilligungsfähigen Prüfungsteilnehmer im Vergleich zur Standardbehandlung seiner Krankheit nur einem minimalen Risiko und einer minimalen Belastung aussetzt“. Für diese nicht unmittelbar eigennützigen klinischen Prüfungen erlaubt die Verordnung strengere nationale Regeln, die in Deutschland in § 40b AMG präzisiert sind und verlangen, dass solche Personen bereits vor Eintritt ihrer Nichteinwilligungsfähigkeit ihre grundsätzliche Bereitschaft zur Teilnahme an nicht eigennützigen klinischen Prüfungen erklärt haben.

Ähnliche Vorkehrungen sieht die Verordnung auch für den Einschluss minderjähriger Prüfungsteilnehmer vor. Allerdings sind hier nationale Sonderregelungen, die strengere Voraussetzungen ermöglichen, nicht vorgesehen.

Im Gegensatz zur Richtlinie beinhaltet die Verordnung nun auch Vorgaben für klinische Prüfungen an schwangeren und stillenden Frauen. Auch hier wird zwischen dem direkten Nutzen für die teilnehmenden Frauen bzw. Embryonen, Föten oder Kinder nach der Geburt und gruppennütziger Forschung unterschieden. Gruppennützige Forschung an dieser Population ist nur zulässig, wenn es aus methodischen Gründen keine Alternative gibt, wenn sie für die Gruppe der Mütter bzw. Ungeborenen von Nutzen ist und für die teilnehmenden Frauen bzw. deren Ungeborene nur ein minimales Risiko und eine minimale Belastung darstellt.

## Sicherheitsberichterstattung

Die Sicherheitsberichtserstattung vom Sponsor an die Behörden erfolgt gemäß Artikel 40 der Verordnung elektronisch an die europäische Datenbank für Pharmakovigilanz (EudraVigilance-Datenbank [10]) und dort in das Modul für klinische Prüfungen (EudraVigilance Clinical Trials Module, EVCTM). Prüfer, sofern sie nicht selbst auch gleichzeitig Sponsoren sind, melden hingegen nur an den Sponsor.

Grundsätzlich zeichnet der Prüfer alle unerwünschten Ereignisse (Adverse Events, AE) auf, außer der (genehmigte) Prüfplan sieht andere Vorkehrungen vor. Der Prüfplan kann bestimmte, als kritisch definierte unerwünschte Ereignisse (Adverse Events of Special Interest, AESI) festlegen, die vom Prüfer innerhalb der im Prüfplan spezifizierten Fristen an den Sponsor gemeldet werden müssen. Schwerwiegende unerwünschte Ereignisse (Serious Adverse Events, SAE), also unerwünschte Ereignisse, die tödlich oder lebensbedrohend sind, eine Hospitalisierung erforderlich machen oder verlängern oder zu bleibender oder schwerwiegender Behinderung oder kongenitalen Anomalien führen, müssen nun innerhalb von 24 h nach Bekanntwerden vom Prüfer an den Sponsor gemeldet werden, sofern im Prüfplan nicht anderweitig bestimmt. Alle mutmaßlichen unerwarteten schwerwiegenden Nebenwirkungen von Prüfpräparaten (Suspected Unexpected Serious Adverse Reactions, SUSARs) müssen vom Sponsor an die EudraVigilance-Datenbank gemeldet werden. Neben SUSARs, die in der klinischen Prüfung selbst auftreten oder nach Ende der klinischen Prüfung noch beobachtet werden, sind auch SUSARs in anderen klinischen Prüfungen des gleichen Sponsors in Drittstaaten meldepflichtig, wenn derselbe Wirkstoff verabreicht wird. SUSARs aus einer klinischen Prüfung mit dem gleichen Wirkstoff eines anderen Sponsors sind dann meldepflichtig, wenn dieser demselben Mutterunternehmen wie der Sponsor der klinischen Prüfung angehört oder gemeinsam mit diesem ein Arzneimittel auf der Grundlage einer vertraglichen Vereinbarung entwickelt. Wie bisher müssen tödliche bzw. lebensbedrohliche SUSARs innerhalb von 7 Tagen erstmalig an die Datenbank gemeldet werden, für alle anderen SUSARs beträgt die maximale Frist 15 Tage.

Zusätzlich muss jeder Sponsor für jedes Prüfpräparat seiner klinischen Prüfungen jährlich einen Sicherheitsbericht an die EudraVigilance-Datenbank übermitteln, solange er klinische Prüfungen mit dem jeweiligen Prüfpräparat durchführt. Sofern es im jeweiligen Prüfplan vorgesehen ist, kann auch ein Sicherheitsbericht für alle Prüfpräparate einer klinischen Prüfung zusammengefasst werden, was sich z. B. für solitäre klinische Prüfungen nichtkommerzieller Sponsoren anbietet.

Sicherheitsmeldungen und jährliche Sicherheitsberichte werden kooperativ von den Mitgliedstaaten bewertet. Details zu dieser gemeinsamen Bewertung wurden in der Durchführungsverordnung (EU) 2022/20 vom 07.01.2022 festgelegt [11].

### Sonstige Meldepflichten des Sponsors

Die Verordnung sieht erstmals auch eine Meldepflicht für schwerwiegende Verstöße gegen die Verordnung selbst oder den Prüfplan vor, sofern durch diese die Sicherheit und die Rechte der Prüfungsteilnehmer oder die Zuverlässigkeit und Belastbarkeit der im Rahmen der klinischen Prüfung gewonnenen Daten „wahrscheinlich erheblich beeinträchtigt“ werden. Für die Meldung eines sogenannten Serious Breach wurde im Dezember 2021 eine entsprechende Guideline durch die EMA publiziert [12].

Für den Fall, dass ein unerwartetes Ereignis voraussichtlich schwerwiegende Auswirkungen auf das Nutzen-Risiko-Verhältnis hat, ergreifen Sponsor und Prüfer dringende Sicherheitsmaßnahmen zum Schutz der Prüfungsteilnehmer und der Sponsor unterrichtet alle betroffenen Mitgliedstaaten über CTIS innerhalb von 7 Tagen nach Ergreifen der Maßnahmen. Daneben besteht nach Artikel 53 seitens des Sponsors eine Meldepflicht innerhalb von 15 Tagen für alle unerwarteten Ereignisse, die sich auf das Nutzen-Risiko-Verhältnis einer klinischen Prüfung auswirken und die nicht als (meldepflichtige) SUSAR klassifiziert sind.

## Transparenzregelungen

Eine wesentliche Forderung an den Gesetzgeber war, die Transparenz in klinischen Prüfungen zu verbessern und wesentliche Elemente einer klinischen Prüfung und deren Ergebnisse früher und besser für die interessierte Öffentlichkeit verfügbar zu machen. Daher wurde in Artikel 81 als Grundprinzip festgelegt, dass alle Informationen über eine klinische Prüfung, die in CTIS gespeichert sind, auch öffentlich einsehbar sein sollen. Davon ausgenommen sind lediglich Inhalte,

- die als Betriebs- und Geschäftsgeheimnisse eingestuft sind; dabei sind der Zulassungsstatus der Prüfpräparate und ein mögliches übergeordnetes öffentliches Interesse an einer (frühzeitigeren) Veröffentlichung zu berücksichtigen,
- die als personenbezogene Daten gemäß der Verordnung (EG) Nr. 45/2001 eingestuft sind,
- die vertrauliche Kommunikation zwischen den Mitgliedstaaten bei der Ausarbeitung des Bewertungsberichts betreffen und
- Inhalte, die der Sicherstellung einer effektiven Überwachung der Durchführung klinischer Prüfungen dienen.

Neben den in der Verordnung genannten Anforderungen bestehen zusätzlich Anforderungen aufgrund der Verordnung (EG) Nr. 1901/2006 über Kinderarzneimittel [13] und den grundsätzlichen „Freedom-of-Information“-Rechten in der EU. In Abwägung der Interessen der Rechteinhaber, in der Regel der Sponsoren einer klinischen Prüfung und/oder der Inhaber von Patenten, wurden 3 verschiedene Kategorien für die Veröffentlichungsregeln in CTIS implementiert (Tab. 2). Grundsätzlich werden Informationen einer klinischen Prüfung in CTIS erst zum Zeitpunkt der ersten Entscheidung über eine Genehmigung öffentlich gemacht und sind dann von den Meilensteinen innerhalb einer klinischen Prüfung und der Kategorie der jeweiligen klinischen Prüfung abhängig, sofern die Sponsoren der jeweiligen klinischen Prüfung eine Verschiebung der Veröffentlichung der jeweiligen Information bei der Antragstellung beantragen [14].

**Table 2:** 

Kategorie	Kategorie 1	Kategorie 2	Kategorie 3
Studientypen	Klinische Prüfungen der Phasen 0 und I sowie sonstige klinische Prüfungen zu Bioäquivalenz, Bioverfügbarkeit oder Biosimilarität	Klinische Prüfungen der Phasen II und III, sofern sie nicht in Kategorie 3 fallen	Klinische Prüfungen der Phase IV und minimalinterventionelle klinische Prüfungen
Datum des Genehmigungsbescheids, Beginn/Ende der klinischen Prüfung, Beginn der Rekrutierung, Unterbrechungen, vorzeitiger Abbruch	Zeitpunkt der Eingabe in CTIS		
Charakteristika der klinischen Prüfung einschließlich der WHO-ICTRP-Datenfelder, Anschreiben und Angaben zu Prüfern und Prüfstellen	Zeitpunkt der Genehmigung im ersten Mitgliedstaat		
	In begründeten Fällen Zurückstellung einiger Inhalte bis zur Veröffentlichung der Zusammenfassung der Ergebnisse beantragbar	–	–
Informationsdokumente für Prüfungsteilnehmer	≤ 7 Jahre^a^	≤ 5 Jahre^b^	≤ 12 Monate^c^
Prüfplan			
Investigator’s Brochure und offene Teile des Dossiers zum Prüfpräparat	Zeitpunkt der Entscheidung über die klinische Prüfung oder der wesentlichen Änderung zur Aktualisierung dieser Dokumente oder der Notifizierung eines aktualisierten Dokuments		
	≤ 7 Jahre^a^	≤ 5 Jahre^b^	≤ 12 Monate^c^
Schlussfolgerung eines Mitgliedstaats zu Teil I des Bewertungsberichts	Zeitpunkt der jeweiligen Entscheidung		
Schlussfolgerung eines Mitgliedstaats zu Teil II des Bewertungsberichts			
Entscheidung über die klinische Prüfung oder eine wesentliche Änderung im Mitgliedstaat			
Ergebniszusammenfassung von geplanten Zwischenanalysen gemäß Artikel 37 Abs. 8	12 Monate nach Zwischenanalyse		
	Fristverlängerung um weitere 18 Monate oder bis zur Erteilung der Zulassung beantragbar	–	–
Ergebniszusammenfassung der klinischen Prüfung einschließlich einer laienverständlichen Zusammenfassung	12 Monate nach Beendigung der klinischen Prüfung, sofern bei klinischen Prüfungen, die für eine Zulassung bestimmt sind, keine längeren Fristen gemäß Artikel 37 Abs. 4 beantragt sind		
	Fristverlängerung um weitere 18 Monate oder bis zur Erteilung der Zulassung beantragbar	–	–

^a^Zeitpunkt der Entscheidung über die Genehmigung im ersten Mitgliedstaat. Der Sponsor kann die Veröffentlichung bis zum Zeitpunkt der Zulassung des Prüfpräparates auf Basis dieser klinischen Prüfung oder bis zu *7 Jahre* nach Ende der klinischen Prüfung aufschieben, je nachdem, welches Ereignis früher eintritt

^b^Zeitpunkt der Entscheidung über die Genehmigung im ersten Mitgliedstaat. Der Sponsor kann die Veröffentlichung bis zum Zeitpunkt der Zulassung des Prüfpräparates auf Basis dieser klinischen Prüfung oder bis zu *5 Jahre* nach Ende der klinischen Prüfung aufschieben, je nachdem, welches Ereignis früher eintritt

^c^Zeitpunkt der Entscheidung über die Genehmigung im ersten Mitgliedstaat. Der Sponsor hat die Möglichkeit einer Verschiebung bis zu dem Zeitpunkt, an dem die Zusammenfassung der Ergebnisse veröffentlicht wird (in der Regel *12 Monate* nach Beendigung der klinischen Prüfung in der EU)

## Erfahrungen und Ausblick

Die Verordnung (EU) Nr. 536/2014 hat das Genehmigungsverfahren klinischer Prüfungen deutlich verändert. Die zu begrüßende vollständige Digitalisierung des Antrags- und Genehmigungsverfahrens hat zumindest in den ersten Monaten nach Inbetriebnahme von CTIS noch einige Schwächen und auch Fehler in der Implementierung offengelegt. Obwohl die technischen Probleme sukzessive behoben werden, hat dies zumindest in den ersten 6 Monaten nach Anwendbarkeit der Verordnung noch zu einer weitgehenden Zurückhaltung bei der Antragstellung über CTIS geführt.

Auch das Verfahren zur Überführung laufender, noch unter der Richtlinie 2001/20/EG autorisierter Studien in das System der Verordnung, das für alle klinischen Prüfungen, die nach dem 30.01.2025 fortgeführt werden sollen, erforderlich ist, warf in den ersten 6 Monaten noch viele Fragen und Probleme auf. Dies als *Transition* bezeichnete Überführungsverfahren ist nicht durch die Verordnung selbst geregelt, sondern lediglich im Fragen-und-Antworten-Dokument der EU-Kommission beschrieben [15]. Letzteres bezeichnet sich selbst bereits als nicht rechtlich verbindlich, so dass insbesondere zu den Überführungsverfahren bei allen Beteiligten weiterhin Unsicherheiten bestehen.

Viele Aspekte, die die Verordnung neu regelt, können derzeit noch nicht abgeschätzt werden. So gibt es im November 2022 noch kaum Informationen zur Praktikabilität der Transparenzregelungen oder zur gemeinsamen Sicherheitsbewertung auf der Basis der neuen delegierten Verordnung (EU) Nr. 2022/22. Trotz dieser Startschwierigkeiten und Unwägbarkeiten ist die Verordnung ein wichtiger Meilenstein in der Harmonisierung, Digitalisierung und Beschleunigung der Genehmigungsverfahren. Ob die neuen zusätzlichen Anzeigepflichten nur den administrativen Aufwand erhöhen oder auch die Überwachung laufender klinischer Prüfungen verbessern, ist derzeit noch nicht sicher einschätzbar. Dennoch ist davon auszugehen, dass die verstärkte Transparenz klinischer Prüfungen und ihrer Genehmigungsverfahren das Vertrauen in das System klinischer Prüfung steigern wird.

## Footnotes

1 GCP-V: Verordnung über die Anwendung der Guten Klinischen Praxis bei der Durchführung von klinischen Prüfungen mit Arzneimitteln zur Anwendung am Menschen.

2 Regelungen zur Verwendung von spezifischen Arten menschlicher oder tierischer Zellen sowie von Arzneimitteln, die solche Zellen enthalten oder aus ihnen gewonnen werden, sowie Regelungen zu Arzneimitteln, die zur Schwangerschaftsunterbrechung dienen oder Suchtstoffe enthalten.

3 Prüfungen, die zwar für den Prüfungsteilnehmer selbst keinen medizinischen Nutzen bringen, aber für die Gruppe, der dieser angehört (z. B. bestimmte Krankheiten oder Lebensalter).
